# Endogenous Retroviruses Function as Gene Expression Regulatory Elements During Mammalian Pre-implantation Embryo Development

**DOI:** 10.3390/ijms20030790

**Published:** 2019-02-12

**Authors:** Bo Fu, Hong Ma, Di Liu

**Affiliations:** 1 Institute of Animal Husbandry Research, HeiLongJiang Academy of Agricultural Sciences, Harbin 150086, China; fubohao810@163.com (B.F.); mahong197400@163.com (H.M.); 2 Key Laboratory of Combine of Planting and Feeding, Ministry of Agriculture of the People’s Republic of China, Harbin 150086, China

**Keywords:** pre-implantation embryo, endogenous retroviruses, zygotic genome activation, epigenetic reprogramming, somatic cell nuclear transfer

## Abstract

Pre-implantation embryo development encompasses several key developmental events, especially the activation of zygotic genome activation (ZGA)-related genes. Endogenous retroviruses (ERVs), which are regarded as “deleterious genomic parasites”, were previously considered to be “junk DNA”. However, it is now known that ERVs, with limited conservatism across species, mediate conserved developmental processes (e.g., ZGA). Transcriptional activation of ERVs occurs during the transition from maternal control to zygotic genome control, signifying ZGA. ERVs are versatile participants in rewiring gene expression networks during epigenetic reprogramming. Particularly, a subtle balance exists between ERV activation and ERV repression in host–virus interplay, which leads to stage-specific ERV expression during pre-implantation embryo development. A large portion of somatic cell nuclear transfer (SCNT) embryos display developmental arrest and ZGA failure during pre-implantation embryo development. Furthermore, because of the close relationship between ERV activation and ZGA, exploring the regulatory mechanism underlying ERV activation may also shed more light on the enigma of SCNT embryo development in model animals.

## 1. Introduction

Transposable elements (TEs), which are the descendants of ancestral viruses, have colonized genomes and now make up about half of mammalian genomes [1,2,3]. TEs can be divided into DNA transposons and retrotransposons [4]. DNA transposons have not yet been characterized during early development, so they are not discussed in this review. In most mammals, retrotransposons are the predominant TEs. They occupy nearly 40% of mammalian genomes [1] and include long terminal repeat (LTR) retrotransposons (also known as endogenous retroviruses (ERVs)) and non-LTR retrotransposons represented by long interspersed elements (LINEs) and short interspersed elements (SINEs) [5,6,7]. The potential transposition activities of unconstrained ERV mobilization can cause insertional mutagenesis or chromosomal abnormality. Therefore, ERVs should be repressed through DNA methylation in terminally differentiated somatic cells, otherwise aberrant activation threatens genomic integrity and results in cancer and autoimmune disorders [8,9]. However, the transcriptional activation of retrotransposons, especially ERVs, is a species-specific and conserved biological process in early mammalian embryos. Transcriptional activation of ERVs induces zygotic genome activation (ZGA) when maternal control gives way to zygotic control, indicating totipotency [10,11,12,13,14,15]. An important question is how ERVs, with limited conservatism across species, mediate conserved developmental processes such as ZGA. Early embryos provide an environment suitable for the transcription of ERVs during epigenetic reprogramming and ERVs may take advantage of developmental epigenomic reprogramming windows to evade silencing by chromatin-modifying enzymes. ERVs, as regulators of gene networks, play multiple critical roles during ZGA. In host–virus interplay, ERV activation is controlled by a multilayered regulatory network that maintains a balance between ERV activation and ERV repression, which results in stage-specific ERV expression during pre-implantation embryo development. The SCNT technique, an asexual reproductive tool, is still inefficient in domestic animal breeding and the establishment of animal disease models. Exploring the molecular properties of ERV activation may also provide clues on the mechanism underlying SCNT embryo development.

## 2. General Survey of ERV Elements in Genomes

Exogenous retroviruses that infect germ cells are integrated into the host genome and inherited by offspring. ERVs derived from exogenous retroviruses are the most abundant transposable elements, accounting for 10% and 8% of the mouse and human genomes, respectively [1,2]. ERVs evolve more rapidly than other TEs, and this has been confirmed by the finding that orthologous ERVs in humans and chimpanzees exhibit signatures of directional selection after the human–chimpanzee divergence that occurred five million years ago [16]. Intact ERV genes encode env, gag, and pol proteins that play key roles in replication and mobilization. These genes are flanked by LTRs that regulate ERV transcription. *Env* encodes env, an envelope protein then enables retroviruses to undergo an extracellular infectious phase. However, most ERVs (e.g., murine endogenous retrovirus-like MuERV-L/MERVL) do not have an *Env* gene and are incapable of horizontal transfer [17,18,19]. *Gag* encodes a specific retroviral antigen and *Pol* encodes integrase, ribonuclease, and reverse transcriptase [20]. ERVs can be divided into class I, class II, and class III elements based on the sequence of their reverse transcriptase gene [1]. The transcription of ERVs is initiated by a 5′LTR promoter, generating a terminally redundant mRNA that is translated into Gag and Gag–Pro–Pol fusion proteins. Then, the ERV mRNA is reverse transcribed into double-stranded cDNA containing the LTR. It is this cDNA copy that is finally integrated into the host genome via ERV integrase [21]. Unlike DNA transposons, ERVs are transcribed into an RNA intermediate that may then be reverse transcribed into cDNA and reintegrated into the host genome at another location. This “copy-and-paste” mechanism tends to increase the copy number of ERVs and thus tends to increase the size of the host genome. However, because of the loss of regulatory elements or protein-coding sequences, most ERVs in mammalian genomes cannot continue with genomic expansion and horizontal transfer [6]. For example, nearly all human ERVs (HERVs) have lost their transposition ability in the human genome [22,23].

Because of ERVs’ viral origin and the history of exposure of hosts to different exogenous retroviruses, genomic ERV content, as genomic parasites, varies significantly between species [6]. Unlike housekeeping activity, the expression of ERVs is species-specific because the LTR elements contain species-specific transcription factor binding sites [24]. ERVs that still have a replication ability rely on the host cell machinery to express their genes. In LTR elements of ERVs that flank the coding sequence of ERVs, *cis*-regulatory sequences and RNA polymerase II promoters are present [25]. During evolution, some ERV elements drift from their original location, whereas other ERVs remain intact, leaving a host genome that is patched with full-length and truncated, active, and inactivated ERVs elements [26]. Following insertion, EVRs often undergo non-allelic homologous recombination between flanking LTRs in *cis* sequences, which results in loss of the coding regions of ERVs, leaving solitary LTRs. Particularly, nearly 90% of HERVs exist in the human genome as solitary LTRs and often contain transcription factor binding sites that are species-specific [2,27]. Thus, transposition also provided an opportunity for genomes to gain novel transcription factor binding sites during mammalian evolution.

## 3. Transcriptional Activation of ERVs Signifies ZGA

Despite the different transcription profiles of ERVs between species, transcriptional activation of ERVs is a conserved event in early embryos [28]. Although ERVs have limited conservation across species, they have the potential to regulate ZGA. Intriguingly, it is not known how such a divergent element can mediate conserved developmental processes such as ZGA. ERVs were identified previously as deleterious genetic elements, and early embryos also employ numerous mechanisms to restrict the retrotransposition of ERVs during development [28]. Paradoxically, ERVs are broadly transcribed into tissue–specific genes or ERV-derived sequences in early embryos and stem cells [10,11,12,29,30,31,32,33,34,35]. The expression of ERVs contributes to the activation of the embryonic genome and to cellular plasticity [32], which is associated with the establishment of totipotency and pluripotency. A large number of ERV–derived sequences are activated, especially in embryonic and cancer cells, and this cell type-specific activation is associated with cell type-specific expression of neighboring genes [36]. MERVL is not expressed in oocytes, but its expression begins to increase after fertilization and peaks at the 2-cell stage, before gradually decreasing until the blastocyst stage [10]. HERV families, such as HERV-H and HERV-K, which are associated with early embryonic development, then signify an undifferentiated state, which indicates that expression profiles of HERV families may herald cell identity [30]. Recently, the high level expression of bovine endogenous retroviruses BERV-K1 and BERV-K2 was also detected in the embryonic blastomeres (2-cell to 16-cell stages) [37]. These suggest that the role of ERV activation is conservative between species. Cleavage stage embryos provide an environment particularly suitable for the transcription of ERVs, where most of the epigenetic markers are wiped off and then reestablished [38]. Remodeling of heterochromatic marks and a relaxed chromatin structure during pre-implantation embryo development provide a time window for the expression of ERVs [39]. The parental DNA methylation and histones are reset across the genome of the zygote, preparing for ZGA and ERV activation. The hydroxylation of 5mC into 5hmC is catalyzed by dioxygenases, the ten-eleven translocation (TET) proteins. The paternal genome undergoes genome-wide loss of DNA methylation via an active mechanism, because TET3-mediated hydroxylation of 5mC accounts for some of the active DNA demethylation of the paternal genome. DNA methyltransferase (DNMT) 1, which methylates hemi-methylated cytosines in CpG sequences, contributes to maintaining genome-wide methylation patterns during replication. Owing to the exclusion of the DNMT1 from the nucleus, the maternal genome is passively de-methylated in subsequent divisions [40]. Hyperaccessibility of chromatin, which is largely determined by histone modifications of its N-terminal tails, is a prerequisite for ZGA. Following fertilization, H3K4me3 and H4 acetylation, which make the male pronucleus permissive for active transcription, are responsible for a minor ZGA [41,42].

After fertilization, maternal inherited transcripts are progressively degraded to give way to the ZGA, setting transition from maternal to zygotic control [43,44]. In mouse embryos, the onset of ZGA has been shown to occur during the 1-cell stage (minor ZGA) [45]. Very weak transcription was observed during the minor ZGA stage [46,47,48]. The 2-cell stage mouse embryos (major ZGA) went through the switch from maternal control to zygotic genome control and were associated with the transient upregulation of many “2C genes” that contain promoters derived from the LTR elements of ERVs [49]. Dramatic transcriptional activation and robust translational activity occur during the major ZGA stage [44,50]. When ZGA failed, embryos failed to develop further [50]; therefore, ZGA may be one of the first critical events during pre-implantation embryo development.

**Figure 1:**
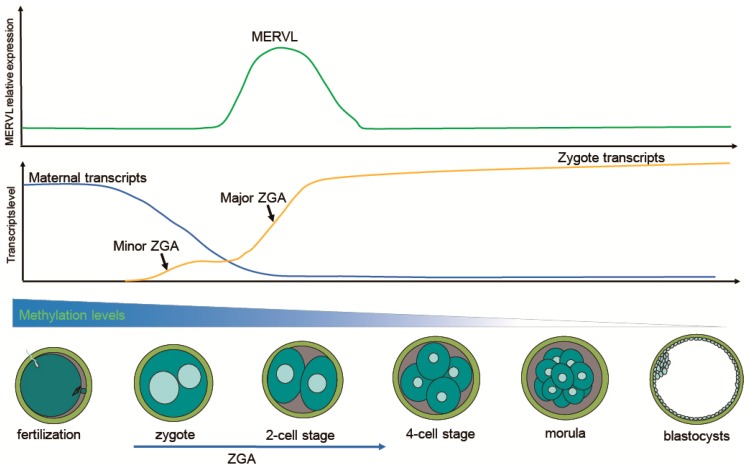
Schematic illustration showing the dynamics of transcription and DNA demethylation during murine pre-implantation embryo development. Shortly after fertilization, embryos undergo extensive global DNA demethylation from the zygote to the blastocyst. Degradation of maternal transcripts is required for ZGA. Minor ZGA occurs at the one-cell stage, while major ZGA takes place at the two-cell stage. Particularly, the expression of MERVL peaks at the two-cell stage, and then gradually decreases until the blastocyst stage.

Transcripts of ERVs that can drive cell transcriptional landscape and developmental stage-specific gene expression, occupy a large portion of the transcriptome during ZGA. For example, ERVs have contributed hundreds of thousands of novel regulatory elements to the human transcriptional landscape [36]. In particular, many critical ZGA-specific genes are regulated by LTR elements of ERVs [11]. Activation of the MERVL gene can be observed as early as 8 h after fertilization (minor ZGA) [10]. Downregulation of MERVL through RNA interference can result in developmental arrest at the 2-cell stage [51]. The transcriptional profile of 2-cell stage mouse embryos is characterized by robust activation of MERVL and 2-cell-specific genes, such as *Zscan4*, *Zfp352*, and *Tdpoz* [52]. In general, the expression of ERVs signifies the onset of ZGA [10]. Several key events during ZGA have been shown in Figure 1.

Although this paper mainly discusses the role of ERV activation in the ZGA process, we shall also bear in mind that ERV-derived transcripts play key roles in trophoblasts and the placenta. Sub-families of ERV proviruses, such as HERV-W and HERV-FRD, are expressed in trophoblasts. This expression is required for cell–cell fusion to enable the formation of syncytiotrophoblasts that are essential for invasive placental development, and the prevention of immune rejection of the fetus at the fetomaternal interface [53]. BERV-K3, belonging to bovine ERVs, is located between interleukin enhancer-binding factor 3 (*Ilf3*) and *Qtrt1* genes on chromosome 7. BERV-K3 has integrated within LOC100848658, from which noncoding RNA could be transcribed. After conceptus attachment to the endometrial epithelium, a high expression of BERV-K3 was detected in the placenta, which may associate with the bovine conceptus epithelial–mesenchymal transition (EMT) and/or its attachment to the uterine epithelium [54].

## 4. ERV Elements Act as Gene Expression Regulators and are Versatile

ERV-derived sequences create alternative promoters during ZGA. LTR elements provide a promoter at the 5′end of ERVs and transcriptional termination and polyadenylation signals at their 3′end. It should be noted that LTRs also contain bidirectional promoters that are able to initiate transcription in both the sense and antisense orientations. For example, in Human T-cell leukemia virus type 1 (HTLV-1), antisense transcription from the 3′ LTR of HTLV-1 regulates the expression of *Hbz*, while sense transcription from the 5′ LTR of HTLV-1 controls the expression of *Tax* [55]. Many ZGA-associated genes were controlled by LTR elements of ERVs. In mice, the LTR promoters of MERVL elements regulate gene networks that are specific to the 2-cell stage of embryonic development, indicating totipotency [13]. A large percentage of ZGA-associated genes are located in proximity to LTR elements [13]. LTR elements contain a binding motif for DUX/DUX4 that is a pioneer transcription factor [24,56,57]. The solitary LTRs that occupy the majority of all HERVs in the human genome still maintain transcriptional and regulatory functions, which affect the expression of neighboring genes [58]. The conserved splice donor site located within LTRs may make LTRs into alternative promoters. During ZGA, these kinds of elements serve as powerful alternative promoters that enable the transcription of neighboring genes, resulting in ‘chimeric’ LTR–host transcripts [11]. Two-cell mouse embryos contain large numbers of chimeric gene transcripts that are identical to mRNA sequences of known host genes, except for the sequence at the 5′end, which is derived from MERVL or other rodent ERV-L elements (e.g., MT2B and MT2C) [11]. In addition, several host genes such as *Tcstv1, Tcstv3, Ubtfl1, Chit1, Eif1a*, and *Zfp352* have transcripts initiating from LTR elements in mouse ESCs, and LTRs have been co-opted by cellular genes as promoters [59].

With pluripotent transcription factor binding sites located within LTRs, the expression of ERVs may also be induced by pluripotent transcription factors. For example, LTR7 that acts as a flanking LTR element in HERV-H contains Oct4, Nanog, Klf4, and Lbp9 transcription factor binding sites and can induce the transcriptional activation of HERV-H, which is a hallmark of naive-like human embryonic stem cells (hESCs) [33].

After fertilization, the assembly of high-order chromatin structure occurs, drastically changing from a condensed maternal and a naive paternal genome to a totipotent state. ERVs provide binding sites for a chromatin organizer, and then participate in the formation of a high-order chromatin structure. For example, CCCTC binding factor (CTCF) regulates the high-order chromatin structure in multiple ways, including participation in chromatin loop formation, connecting the long-range enhancer-promoter, and insulating epigenetic spreading [60]. Binding sites of CTCF are enriched in ERV elements [61]. When bound by CTCF, ERV elements can organize a high-order chromatin structure, thus promoting the global remodeling of chromatin architecture during pre-implantation embryo development [62,63].

**Figure 2:**
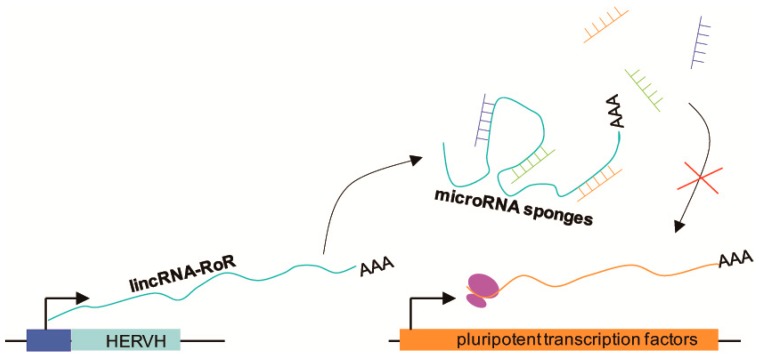
Schematic illustration showing how pluripotent transcription factors are protected from miRNA-mediated degradation. LincRNA-RoR, a long intergenic noncoding RNA, is transcribed from human endogenous retrovirus subfamily H (HERVH), and then acts as an miRNA sponge to prevent miRNA-mediated degradation of pluripotent transcription factor mRNAs. The intact pluripotent transcription factor mRNAs can then be translated.

ERV-derived long noncoding RNAs (lncRNAs) participate in the control of pluripotency. Biomarkers of pluripotency, such as Oct4, Sox2, and Nanog, promote the expression of ESC-specific genes and suppress differentiation [64,65]. In posttranscriptional networks, microRNAs (miRNAs) act as posttranscriptional modifiers and contribute to restraining pluripotency. For example, miR-145 represses Oct4, Sox2, and Klf4 by binding to their 3′UTRs, and miR-134, miR-296, and miR-470 target the coding DNA sequence of Nanog, Oct4, and Sox2 [66,67]. LincRNA-RoR is a long intergenic noncoding RNA derived from human endogenous retrovirus subfamily H (HERVH) [68]. LincRNA-RoR acts as a competing endogenous miRNA sponge and protects pluripotent transcription factors from miRNA-mediated degradation; therefore, it is necessary and sufficient to maintain pluripotency and control ESC differentiation [69] (Figure 2). Jens [70] found that the lncRNA HPAT5 can also function as an miRNA sponge for the let-7 miRNA family to positively regulate pluripotency in ESCs. In addition, Fort et al. [31] found numerous pluripotent lncRNAs that contained unannotated antisense, intergenic, and intronic transcripts derived from ERV elements. Particularly, many stem cell-specific transcription start sites are not associated with protein-coding genes, but with these kinds of ERV elements in mice and humans.

The activation of ERVs may contribute to genome defense. LTR5HS, a subclass of HERV-K, is transcribed from an LTR at the 8-cell stage (ZGA in human embryos) and contains the Oct4-binding motif. Grow et al. [35] found that by binding to LTR5HS, Oct4 drove the expression of HERV-K proviruses, producing viral-like particles and Gag proteins in pre-implantation embryos. Then, the overexpression of Rec, HERV-K accessory protein, is sufficient to increase the level of virus restriction factors such as IFITM1 during this process and contributes to fighting against exogenous viral infections.

## 5. Regulation of ERV Activation

How exactly species-specific ERVs are activated to function as gene regulators in host cells and, conversely, how host cells defend themselves against ERV activation during the window of epigenetic reprogramming (pre-implantation embryo development) to prevent widespread retrotransposition, has been investigated. ERV transcripts are under acute surveillance by multilayered and interleaved systems that ensure a subtle balance between ERV activation and ERV repression, resulting in stage-specific ERV expression during pre-implantation embryo development.

Unlike differentiated somatic cells with high DNA methylation levels, pre-implantation embryos undergo DNA methylation reprogramming, which is associated with establishment of the pluripotent state [71]. TET proteins catalyze oxidation reactions that convert 5-methylcytosine (5mC) to 5-hydroxylmethylcytosine (5hmC), or further to 5-formylcytosine (5fC) and 5-carboxylcytosine (5caC) [72,73,74,75]. The activity of TET proteins in a pre-implantation embryo allows ERVs to evade DNA methylation-mediated transcriptional repression. In response, numerous histone modifications predominate in silencing ERV expression in pre-implantation embryos and ESCs where DNA methylation mediated silencing is compromised [28,75,76]. Acquiring H3K9me3 while losing DNA methylation in LTR loci is a mark of epigenetic transformation [77]. H3K9me3 is enriched at the LTRs of many class I and class II ERVs in mouse ESCs (mESCs) [78]. Histone methyltransferase SETDB1 catalyzes the addition of methyl groups to H3K9 [79]. When SETDB1 was knocked out from mESCs, significant upregulation of several class I and class II ERVs was detected [80]. Correspondingly, removing KRAB-interacting scaffold protein KAP1, an SETDB1-interacting protein, also resulted in upregulation of the same ERVs in mESCs [80,81], indicating that KAP1, acting as a SETDB1–KAP1 complex recruiter, is also required for silencing ERVs [82]. KAP1 or its partner SETDB1 can repress several classes of ERVs in ESCs, but not in embryonic fibroblasts, because DNA methylation takes over control of the ERVs in differential somatic cells [81,83,84].

Indeed, KAP1-mediated repression is sequence-specific, because KAP1 is recruited to repetitive sequences through site-specific Kruppel-associated box zinc-finger proteins (KRAB-ZFPs), the largest transcription factor family in mouse and human genomes. KRAB-ZFPs are characterized by the N-terminal KRAB domain and a tandem array of C2H2 zinc fingers [28,76]. KAP1 represses multiple classes of ERVs, including intercisternal A-type particles and MERVK in mice or HERVK in humans, which indicates that ERVs may recruit KAP1 through different site-specific KRAB-ZFPs [85,86]. This hypothesis is supported by the observation that KRAB-ZFPs encoded from host genomes are species-specific and rapidly evolving, corresponding to the rapid evolution of viral sequences [87,88,89,90,91]. ERVs and the host genome are often referred to as an evolutionary arms race [92,93]. The continuous cycle of KRAB-ZFP evolution against ERVs may provide a driving force for new adaptations in mammals [83]. For example, the expansion of the *Znf91* subfamily across primate lineages reflects KRAB-ZFP adaptive evolution [94].

Unlike class I and class II ERVs, the repression of class III ERVs, including MERVL elements, is KDM1A (H3K4 demethylase)-mediated. MERVL sequences or LTR elements are targets of KDM1A repression. MERVL elements display increased methylation of histone H3K4, increased acetylation of H3K27, and decreased methylation of H3K9 in *Kdm1a* mutant ESCs. Therefore, significant upregulation of MERVL elements and their flanking LTRs occurs in *Kdm1a* mutant ESCs [59].

**Figure 3:**
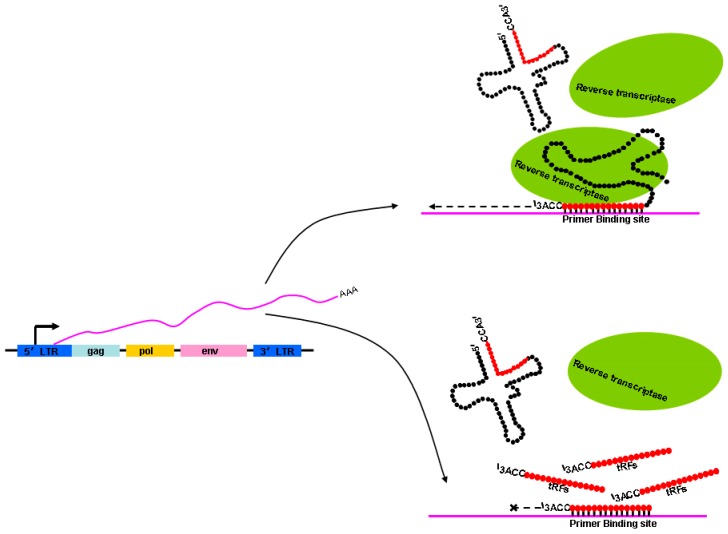
Schematic illustration showing how the reverse transcription processes of endogenous retrovirus (ERV) transcripts are repressed. The ERV genes *env*, *gag*, and *pol* are flanked by LTRs that regulate ERV transcription. The 3′terminus of intact mature tRNA is used as the special primer to complete the reverse transcription process. However, the process is interrupted when 18-nt 3′-tRF (tRNA-derived fragment) binds to the primer binding site.

In addition to numerous pathways based on histone modifications, other mechanisms may function to control ERV activation. LTR retrotransposons use mature tRNAs that are selectively packaged into the intracellular virus-like particles (VLPs), and then bound by primer binding sites of the viral RNA as primers for reverse transcription, thus initiating the synthesis of minus-strand cDNAs [95]. Using the 3′terminus of mature tRNAs, the special primer for reverse transcription, to bind the primer binding sequence in ERV transcripts, ERVs start-up the reverse transcription process. However, tRNA-derived fragments (tRFs) that are 18-nt long can strongly inhibit the reverse transcription processes of ERV transcripts when 18-nt 3′-tRF binds to the primer binding site. Namely, 18-nt 3′-tRFs and mature tRNAs compete for primer binding sites at the same time [96] (Figure 3). In addition, the transposition activity of ERVs is inhibited by 22-nt 3′-tRFs that degrade the protein-coding mRNA of autonomous ERVs through post-transcriptional silencing with miRNA-like mechanisms that tolerate mismatch [88]. The 18-nt 3′-tRF and 22-nt 3′-tRF both contain a 3′-CCA at the end of their tRNA precursors, indicating that they both originated from mature tRNAs. A rapid increase in tRFs at the 8-cell stage was found in mouse pre-implantation embryos [97]. Particularly, tRFs are abundant in mature sperm [98,99] and can be delivered to a pre-implantation embryo through fertilization [100]. Therefore, tRFs derived from sperm may also inhibit the retrotransposition activity of ERVs in pre-implantation embryos where the genome lacks epigenetic markers such as DNA methylation and histone modification [101].

Homeodomain is a DNA-binding motif present in transcription factors. Double homeodomain-containing DUX-C proteins, located at the top of a transcriptional hierarchy, are expressed before the ZGA phase in placental mammals [24,102,103]. The human DUX-C homologue is DUX4, and the mouse DUX-C homologue is DUX. After fertilization, a globally transcriptionally permissive state caused by the loosening of chromatin and increased histone mobility may induce DUX-C activation [104,105]. In mammals, DUX-C family homologues (*DUX-C*, *DUX4*, and *DUX*) show a microsatellite tandem-array organization that, thus, has high copy numbers in the genomes [106]. The weak transcriptional activation of each *Dux-c* gene copy will result in a surge of the DUX-C protein. The two separate DNA-binding domains in DUX-C orthologues could provide an enhanced and synergistic binding ability. DUX4 can also bind at DNase I inaccessible sites, and then acetylate histone H3 at lysine 27, to open up chromatin via recruiting p300/CBP [107]. All these permit DUX-C orthologues to function as pioneer regulators of ZGA. DUX4, which is known to be associated with facioscapulohumeral dystrophy (FSHD), activates a large portion of the genes normally expressed at the onset of ZGA, particularly including cleavage-specific genes that have an upstream enriched binding motif for DUX4 [108]. When overexpressed in human-induced pluripotent stem cells, DUX4 also activated ZGA-related genes and retrotransposons, particularly HERV-L. Similarly, the expression of DUX peaked in 2-cell stage embryos (ZGA in mouse), and overexpression of DUX in mouse ESCs also activated ZGA-related genes. Deleting DUX in mouse zygotes caused ZGA failure and impaired developmental progression [56]. DUX upregulation also appeared in 2C-like cells that were identified by the reactivation of ERV elements such as MERVL, which then drives the transcription of many ZGA-related genes in embryos [13]. Together, the above phenomena indicate that DUX-C-family homologues serve as pioneer transcription factors that seem to drive the expression of ZGA-related genes such as *Zscan4* and regulate ERV activation, including MERVL. Furthermore, because of the versatility of ERVs, their control by DUX proteins strengthens the transcriptional regulation potential of DUX in return.

Although mouse DUX and human DUX4 can both activate orthologous genes such as *Zscan4* in mouse and human myoblasts respectively, the overexpression of human DUX4 did not activate ZGA-related murine MERVL LTRs [57]. The species-specific transcription factor binding sites located within LTRs elements and the more divergent homeodomain (the first homeodomain) in DUX-C family proteins means that the activation of ERVs by DUX is species-specific, although DUX proteins still exhibit functional conservation because DUX-C family proteins have a more conserved homeodomain (the second homeodomain) [57]. The ancestral DUX proteins can regulate embryonic transcription conservatively, whereas the divergence of the DNA-binding motif of DUX-C family proteins indicates the adaption to species-specific activation of ERVs during evolution [24].

Expression of MERVL and activation of 2-cell stage genes induced by DUX are rapidly repressed after the 2-cell stage. Therefore, embryos exit the 2-cell state, otherwise ZGA will fail and embryonic development will be blocked [59,104]. LINE 1 is expressed throughout murine pre-implantation development [109]. In addition, human transposable elements such as

**Figure 4:**
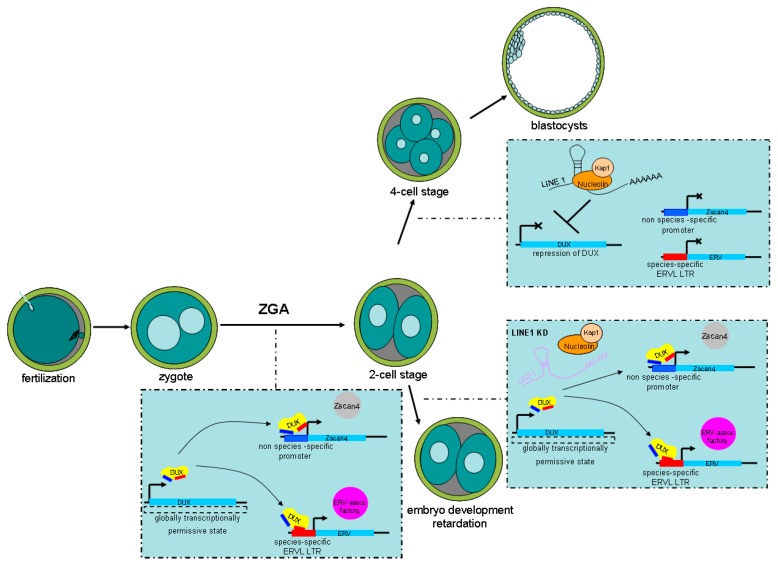
Schematic illustration showing how endogenous retrovirus (ERVs) activation is controlled by double homeobox DUX during murine pre-implantation development. After fertilization, a globally transcriptionally permissive state caused by the loosening of chromatin activates DUX. Then, DUX drives the expression of zygotic genome activation (ZGA)-related genes such as *Zscan4* and regulates ERV activation. LINE 1 RNA represses DUX by recruiting Nucleolin/Kap1, thereby indirectly repressing ZGA-related genes and ERV elements. This allows 2-cell embryos to develop into the 4-cell state. In contrast, LINE 1 knockdown (KD) causes persistence of the 2-cell state and failure of ZGA.

LINE 1 were also detected in human pre-implantation embryos [110]. Further research demonstrated that LINE1 knockdown caused persistence of the 2-cell state and failure of ZGA in murine pre-implantation embryos. In addition, LINE 1 RNA served as a nuclear RNA scaffold that recruited Nucleolin/Kap1 to repress DUX; thus, indirectly repressing the transcription of ERVs. LINE 1 depletion leads to failure to repress DUX/2-cell programing, which then leads to defective ZGA, resulting in embryo development retardation. Therefore, persistent LINE 1 RNA may be required for the repression of DUX and its associated ERV elements during pre-implantation embryo development [111] (Figure 4).

## 6. Insights into Improving the Developmental Potential of SCNT Embryos

During somatic cell nuclear transfer (SCNT) reprogramming, differentiated somatic nuclei were transferred into enucleated oocytes and the reconstructed embryos acquired totipotency to produce viable cloned animals [112,113]. Although many mammalian species have been successfully cloned from various somatic cells, the SCNT technique is limited by its low efficiency because differentiated somatic cells often retain somatic chromatin states, even in reconstructed embryos [114]. In mouse SCNT embryos, nearly half of them display developmental arrest during pre-implantation embryo development [115]. The gene expression profiles of the somatic cells are reprogrammed to those of the 2-cell mouse embryos during the ZGA process. However, abnormal gene expression profiles were detected in early SCNT embryos, particularly in ZGA-stage SCNT embryos [116,117]. Besides mice, developmental defects have also been detected at the time of ZGA in other cloned species [118,119,120]. Unlike mouse embryos, ZGA occurs at the 8-cell stage in bovine embryos [121], which may give bovine embryos a longer time for ZGA-associated genes to be reprogrammed and activated sufficiently. Indeed, the developmental rates of cloned bovine embryos, which are similar to those of IVF bovine embryos [122], are relatively higher than the developmental rates of other cloned species [114]. It has been postulated that ZGA is necessary for somatic cell reprogramming [123]. Insufficient reprogramming and failure of ZGA are common defects of SCNT embryos, which impairs the final developmental potential of SCNT embryos [118,119,120]. ERVs such as MERVL can be activated specifically during ZGA [51,124,125,126,127]. With LTR elements serving as alternative promoters, ERVs drive the expression of hundreds of chimeric transcripts [11]. Therefore, the expression of ERVs is a hallmark of the ZGA process. However, compared with fertilized embryos, MERVL was downregulated at the 2-cell stage in the cloned mouse embryos. Furthermore, ZGA-associated genes such as *interferon-γ*, *Dub-1*, and *Spz1* were also inhibited in SCNT embryos [128]. The red fluorescent protein tandem dimeric tomato (tdTomato) reporter under the control of MERVL-LTR (MERVL:tdTomato) has been used to monitor ZGA events in real time [13]. Using transgenic mouse lines as the source of donor cells and sperm that contain the MERVL::tdTomato reporter, the expression pattern of ERVs such as MERVL can be observed in early SCNT embryos and intracytoplasmic sperm injection (ICSI) embryos in real time. Only 12% of SCNT embryos exhibited the reactivation of somatic MERVL::tdTomato during ZGA, whereas 92% of ICSI embryos exhibited the reactivation of MERVL::tdTomato during that stage [129]. Moreover, single-cell RNA-seq data also indicated that ZGA-associated genes could not be properly activated in tdTomato-SCNT embryos compared with normal fertilization embryos [129]. Given the key roles played by ERVs during ZGA, downregulated ERV expression may cause the failure of ZGA, leading to defects of SCNT embryo development.

The failure of ERV expression during ZGA in SCNT embryos may be induced by preexisting epigenetic barriers in the genomes of differentiated donor cells. Matoba et al. [130] found that ERVs are suppressed in SCNT embryos compared to IVF embryos and identified hundreds of reprogramming resistant regions (RRRs) in SCNT embryos. These RRRs, where ERV elements such as LTRs are located, are gene-poor regions and are specifically enriched with H3K9me3. H3K9me3-initiated heterochromatin assembly may prevent the activation of ERV elements in RRRs. In general, the conserved epigenetic markers that originated from differentiated donor cells preexisted in RRRs. Unfortunately, conserved epigenetic barriers impede the expression of ERVs and ZGA during SCNT reprogramming. Elimination of epigenetic barriers located in ERVs of RRRs may improve ZGA and SCNT efficiency. For instance, the ectopically expressed H3K9me3 demethylase Kdm4d can remove the inhibitory chromatin state in RRRs, thereby improving ZGA and SCNT efficiency [130]. In another case, Yang et al. [129] found that enrichment of H3K27me3 was also associated with the failure of ERV activation. KDM6A and KDM6B are H3K27me3-specific demethylases that are functionally redundant and compensate for each other, so a decrease in KDM6A or KDM6B expression is accompanied by an increase in KDM6B or KDM6A expression, respectively. H3K27me3 levels decrease when injected with KDM6B small interference RNA (siRNA), which increased KDM6A expression and correspondingly increased MERVL levels. Finally, KDM6B siRNA injection improved the developmental potential of SCNT embryos by increasing ZGA-related ERV expression.

## 7. Conclusions

ERVs behave like retroviruses and play a variety of functions. The subtle balance between the activation and silencing of ERVs during pre-implantation embryo development implies a multilayered regulatory network is involved in host–virus interplay. An increase in ERV expression at specific developmental stages marks key events such as ZGA, and ERV activation can be inhibited by various mechanisms, such as DNA methylation, histone modification, post-transcriptional silencing, and transcription factor inactivation. From an evolutionary perspective, ERVs and the host genome are often regarded as being an arms race that is embodied by KRAB-ZFP adaptive evolution. ERVs and the host genome also have a cooperative symbiotic relationship that supports the amplification of ERVs in the host genome and provides the regulatory elements in gene networks. In particular, specific ERV activation (also known as the marker of ZGA) provides clues to elucidate the mechanism underlying SCNT embryo development. Although, due to ethical and technical constraints, regulating ERV activation should not be applied in clinical practice, ERV activation may be a key developmental event across species. Therefore, precisely regulating ERV activation may provide a new perspective for investigating the molecular mechanism underlying the ZGA process and improve the developmental potential of SCNT embryos in model animals.
